# Sexual Exhaustion (Neurasthenia)

**Published:** 1880-05

**Authors:** George M. Beard

**Affiliations:** Fellow of the New York Academy of Medicine; Vice-President of the American Academy of Medicine; Member of the American Neurological Association, etc., etc.


Vol. I.——*May, 1880.*——No. 5.

ARTICLE I.

### THE SYMPTOMS OF SEXUAL EXHAUSTION (SEXUAL NEURASTHENIA).

BY GEORGE M. BEARD, A. M., M. D.

Fellow of the New York Academy of Medicine; Vice-President of the American Academy of Medicine; Member of the American Neurological Association, etc., etc.

In a series of papers published at different times during the past eleven years I have endeavored to describe in detail the symptoms of neurasthenia, or nervous exhaustion in general, and to indicate the differential diagnosis between this disease and anemia, hysteria, hypochondria, and other diseases or symptoms of disease with which it is often confounded. As the disease neurasthenia is now, after a tolerably long delay, becoming recognized by neurologists and general practitioners, it seems to be proper to take a step farther and study the special phase of neurasthenia to which the term *sexual neurasthenia*, or sexual exhaustion, may be applied. One cannot study neurasthenia in either sex very long without becoming convinced that the reproductive system is a prominent and, in many cases, the leading factor in causing and in maintaining this disease. Certain verifiable facts are quite instructive on this subject.

First of all, neurasthenia is most frequent and most severe during the period of sexual activity, between the ages of fifteen and fifty or sixty. It exists before fifteen and after fifty or sixty—even in extreme age, but it is much more rare and less distressing. Secondly, there are certain symptoms in neurasthenia as it appears between fifteen and sixty which are not common in neurasthenia as it appears in youth and age. What these symptoms are will be explained in this essay.

The subject is restricted to sexual exhaustion as it exists in the male, for the reason that the symptoms of neurasthenia as it exists in females are, and for a long time have been, understood and recognized even by those who have never used or heard of the term neurasthenia. Very frequently a mistake is made in the treatment of these forms of female exhaustion, constitutional being almost displaced by local measures; but the condition is, by the best physicians, suspected from the patient's history before any examination has been made, and no attempt at treatment is made until there is a full diagnosis of the condition of the reproductive organs. Cases of sexual exhaustion in the male, on the contrary, are not suspected from the history given by the patient; no examination is made, and no inquiry into the sexual history or hygiene; it is not known what symptoms are to be regarded as indicative of genital disturbance or complication, and the general result is that these cases, which are very common, indeed fully as common, I think, as analogous cases in females, are dismissed as hypochondriacs.

The symptoms of sexual neurasthenia are local and general. Many of the local symptoms are sufficient of themselves to suggest the disorder of which they are the results; but this is not true of all of them. The general symptoms are none of them diagnostic; it is only by studying a large number of these symptoms singly, and in their relation to each other and in connection with the various local symptoms, that we are able to reach a sure diagnosis of sexual exhaustion in any case. In this, as in most nervous diseases, pathognomonic symptoms are rare. It is not necessary that all, or even a majority, of these symptoms shall be found in any one case to establish the diagnosis beyond question; some patients will present but a few; others, many of the local or constitutional phenomena; but by rightly reading even a limited number of these signs there will usually be no more severe difficulty in arriving at a clear result than in diagnosticating any other disease of the nervous system.

I begin with the local symptoms, giving especial prominence to those that are least known and, therefore, least likely to be observed. Some of these local symptoms, it may be noted at the outset, are found in urethral stricture, in contracted meatus and in prostatitis, being excited by these diseases, sometimes directly, sometimes indirectly.

I. *Various Stages of Impotence and Spermatorrhœa.*—The extreme stages of these morbid phenomena are so well known that it is not needful to more than refer to them for the sake of completeness. The milder, subtler and more evanescent degrees of these affections are, however, but rarely thought of or appreciated. Impotence is a symptom of very wide range and gradations, beginning with premature emission, or simply waning pleasure in the sexual act, and advancing through the stages of ejaculation before intromission, deficient desire and power, to absolute want both of desire and power. Similarly, involuntary emissions may be so rare and have so little effect on the health as to be practically physiological, or may occur several times nightly, or even in the day. They may or may not be complicated with true spermatorrhœa, that is with the flowing away of semen in the urine, or at stool, or on excitement. The existence of even quite frequent involuntary emissions by night or day is not a proof of the existence of true spermatorrhœa. There may be very frequent emissions and yet no spermatozoa in the urine or any discharges at stool; on the other hand emissions may be infrequent, as rarely as once or twice monthly, and yet spermatorrhœa may be active all the time; indeed it would appear that the spermatorrhœa acts as a relief for the accumulated seminal fluid and saves the nocturnal discharges; thus it happens that patients are often deceived; they observe that their emissions are less frequent, and suppose that they are recovering, when really the fluid is but taking another mode of exit.

Only the microscope can answer the question whether spermatorrhœa does or does not exist; all conclusions formed from the various local or general symptoms may be swept away by careful and repeated microscopic examinations by an expert with that instrument. As I have elsewhere shown, true spermatorrhœa, though not so common as the charlatans seek to prove, is yet far more common than is admitted by the medical authorities; it is not found because it is not looked for, and usually not even suspected; those who have the symptoms and history that ought to make us examine the urine or the discharges at stool, are little inclined to consult their physicians, or if they do so, they are dismissed as hypochondriacs, and there is no thought of making any other diagnosis. In some cases this snap judgment is wrong; the patient is really suffering, not in mind alone, but in body. In the majority of cases where trouble with the genital system is suspected by patients, some form of trouble does exist. It may not be what the patient suspects, it may not be so grave as he has fancied, but there is usually something abnormal that requires treatment or hygiene; and there is also need of sound instruction on the whole subject of the management of this function. There is, according to my observation and experience, far more of hypochondria (that is, groundless fear of disease) in regard to the heart than in regard to the reproductive system. A woman may be mistaken in suspecting that all is not right with the womb, but in the majority of instances of suspected difficulty of that organ there is some disorder, either of that or of some other part of the reproductive apparatus; while there are very many women who remain long invalids from uterine or ovarian trouble without the fact ever being suggested by herself or by her adviser. In men as in women, the removal through treatment and hygiene of the real disease of the genital system removes the morbid fear of disease that does not exist.

The long disputed question whether seminal discharges take place from the penis during or directly after stool, and in some instances during any form of sexual excitation, the microscope answers in the affirmative. In many instances, perhaps in the majority, certainly in the majority of those discharges that take place during erotic excitement, the fluid discharged comes from the prostate, or from Cowper's glands, or from both; but in a certain proportion of cases it is just as demonstrable that the spermatic fluid comes out at such times. There is some considerable difficulty in getting a specimen of the fluid that appears under such circumstances; the quantity is small, and if preserved soon dries. Hence the most satisfactory examinations have been made by the sufferers themselves when they had the fortune to be experts with the microscope. In this, as in the flow of spermatozoa in the urine, the microscope is absolutely indispensable, inasmuch as the appearance or the quantity of the discharge furnishes, according to my observations thus far, no sure guide. Patients often suppose that urine is spermatic, judging from the color, or indeed without examining it at all; they are not aware that at least four or five different secretions are discharged from the penis. In some cases they are wrong in these suspicions, in others they are quite right. It is for the physician to decide each case by special study.

In relation to this branch of the subject it should be noted here that all these phases of impotence and involuntary emissions and true spermatorrhœa, nocturnal and daily, occur in full force in the married as well as in the single. The notion that silly and imaginative young men are the only sufferers from these disorders is a mistaken one; and it is a serious mistake to make this assumption, for it keeps us from searching for a right diagnosis, and interferes with the scientific study of the disease. Some of the very worst cases of these troubles I have ever seen have been in the happily married men who, as a result of previous or present excesses in the natural or unnatural way, have brought themselves into the condition which is supposed to be the special and peculiar property of the young and unwedded. Under the inspiration of this error these cases are often advised and urged to marry—the very step which, in some instances, they should not take until they have been brought into a better condition, when not unlikely a judicious marriage will properly supplement the effects of medicine and hygiene. True spermatorrhœa is the cause of many obscure symptoms that are often referred to the stomach or liver.

II.—*Severe Itching of the Perineum, Scrotum and Root of the Penis.*—This symptom has been noticed by me in a number of cases. That it is a part of the genital difficulty is proved by the fact that it co-exists with other symptoms hereafter to be described, and is quickly relieved by general sedative treatment without any local applications. This symptom is a true pruritus—itching without any objective appearance to account for it. The itching is usually worse at night on retiring, but it is often troublesome during the day. Temporary alleviation of this pruritus may often, if not in all cases, be obtained by the application of strong anti-pruritics, such as a mixture of chloral, camphor, morphine, peppermint, and aconite and chloroform; but the most substantial results come from the treatment of the condition of which it is the symptom.

III.—*Anesthesia or Numbness of One-half of the Penis with Coldness of the Parts.*—This anesthesia is more likely to be on the left side, and may be detected by an examination with the electric brush or with the esthesiometer. As I have elsewhere stated, there appear to be cases where there is electro-anesthesia—that is, anesthesia to the faradic current, but not any demonstrable diminution of the sense of touch. I am not sure whether this condition exists in penis. The coldness of the parts which may or may not accompany the anesthesia needs no thermometer for its detection; it can be felt by the patient and by the hand of the physician. The glans penis and the scrotum are the most likely to exhibit this phenomenon.

IV.—*Pain on Coition or Urination.*—Sometimes one of the earliest signs of excess, and of the debility that follows excess, is slight burning pain during or right after emission or urination. This pain may last several moments, or longer, after the act is completed, and may extend to the perineum as a form of neuralgia. A most remarkable case of this kind has lately been reported by Dr. J. B. Sullivan, of Stanton, Michigan. A bride of four weeks standing confessed that her husband at the point of the culmination of the orgasm is seized with a numbness and with loss of all pleasure in the penis, and this is at once followed by severe pain, which the slightest motion or friction intensifies so that he is unable to separate until the penis has become quite flaccid. Sometimes an hour or more, according to the bride's statement, would pass before separation would be possible. This quick alternation of anesthesia and hyperesthesia during the sexual act is, I believe, quite exceptional. It would be interesting to know the habits and condition of the husband prior to marriage, and what other symptoms attended this peculiar phase of sexual incompetence. Urination is sometimes accompanied with somewhat of the pleasure of seminal emission.

V.—*Pain and Heaviness in the Perineum and Prostatic Region.*—A feeling of weight is often spoken of by patients, as though they had been struck in the perineum or a hard body were in the rectum. Usually this sensation appears to be connected directly with an irritated and congested prostate, but there is no evidence in the majority of cases that there is any enlargement of that organ. Sometimes this sensation extends toward the scrotum, and various and hard-to-be-defined sensations radiate over the buttocks and thighs on the most trifling excitement. With some these abnormal feelings are noted only when there has been excess, or at least some excitement.

VI.—*Urethral Hyperesthesia.*—The most common seat of an excessive sensibility of the urethra is in the prostatic region; but in the anterior portion, also, spots or districts of abnormal sensitiveness are noticed, and the entire canal may be like a bare nerve. Cases of sexual neurasthenia are not very infrequent where the urethra at or near the meatus is so sensitive that a small and well-oiled sound cannot be passed for an inch without causing distress. One of my medical patients, a married man with a family, but with a long array of nerve symptoms connected with genital debility, was an hour passing a sound of moderate size on himself, so great was the hyperesthesia. I have seen a number of cases where I could do nothing in the way of local treatment by sounds, or even by electricity, until this excessive sensitiveness had been subdued by medication with sedatives; and I have seen two or three cases where local internal treatment was for this reason impossible, or where it did more harm than good, however cautiously used; instruments would not be tolerated. Reasoning from analogy of what the oculists find in the eye in hyperesthesia of that organ connected with sexual disorders, it is quite probable that if we could examine the whole track of the urethra when in a hyperesthetic state we should find a passive congestion—possibly a slight venous dilatation, not enough congestion to account for the symptoms, but yet of a certain pathological interest. In some cases we see through the endoscope a decided redness and swelling. This urethral sensibility is not constant, it may come and go like all the symptoms of the neurasthenic state.

VII. *Congestion of the Lips of the Meatus.*—In quite a number of cases of disorder of the reproductive organs, whether associated or not with general neurasthenia, I find a redness of the lips of the urethra. This is not found in perfect health, and it is not universal in neuro-genital disorders, but in a certain percentage of cases it is easily demonstrated, varying much at different times. I have been accustomed to refer this appearance to prostatic congestion and irritation, and have supposed that the lips are affected by sympathy, and reveal the state of the prostate just as the tongue reveals the state of the stomach.

VIII. *Irritable Prostate without Evidences of Prostatitis.*—Irritability of the prostate is indicated by the sensations of weight and heaviness before mentioned, and it is the one cause, perhaps the chief cause, but not the only cause, of such sensations. The diagnosis of irritable prostate is suggested when in addition to these symptoms of perineal uneasiness we have disorders of urination hereafter to be mentioned, and tenderness on pressure when an examination is made through the rectum. An irritable prostate is analogous to irritable spine, breast, ovaries and testes.

One very marked instance of this I recall where for many years the patient had been kept in a kind of invalidism by this condition of the prostate. One of the very best surgeons in this department had carefully studied his case, and had decided that there was no important enlargement or other recognizable malady of the prostate beyond irritation, and recommended a very long residence and travel abroad. This advice, which was impracticable, was given on the supposition that the patient was suffering from functional nervous disease only. My own subsequent examination of the case confirmed this diagnosis, and the after history and the results of the treatment yet further confirmed it. In this case the irritation was so great that the patient could at no time walk far—rarely beyond a mile or so—without experiencing unpleasant local fatigue.

A phase of genital debility that is not so well-known is that of aspermatism, lack of seminal secretion, or difficulty in ejaculating the semen, even while the erectile power is normal, or perhaps excessive. It is supposed that spasmodic contraction of the walls of the ejaculatory ducts may be the philosophy of this disorder; or it may be the actual want of the seminal fluid in the vesicles. It is improbable that there would be desire and power of erection when no fluid was secreted by the testicles.

IX. *Prolonged Coition.*—In this same line of difficulty is very prolonged coition without emission, or before the emission takes place. This is just the opposite of too early emission, which is a far more familiar form of weakness of this function. The law of opposites in functional nervous disease is, I may here remark, very interesting indeed; it is, however, no more observable in maladies of the reproductive system than in maladies of other parts, and in the general symptomatology of diseases of the nervous system.

By a degree of practice such as is said to have been carried out in one of our socialistic organizations it is possible to *prolong* intercourse for half an hour, or even an hour, without an emission; and there are those who keep up this practice for years, though in the majority of cases they are injured by it sooner or later. Investigations that I once instituted convinced me that this habit had on the whole an injurious effect on the members of the community where it was practiced; both sexes were less vigorous and strong in appearance than is normal to persons of the race and social condition to which they belong. Excitement of this kind, protracted and ending without normal gratification, is a powerful strain on the nervous system, and it is proof of the immense reserve force in the nervous system that indulgence of this sort is so slow in producing its evil effects. Two cases have been brought to my notice where this habit was maintained for many years before any bad effects were noticed ; and in both instances it was the man who was injured. The injury was functional, however, and improvement at once followed a change in the hygiene.

There are individuals who, partly as a result of positive objective debility of the generative apparatus, and partly also as a result of excitement and over-anxiety, fail in the sexual act through this very prolongation of intercourse; instead of coming too soon, as is so frequent in such cases, the emission comes very late or not at all. This symptom alarms patients, but it is not necessarily a very bad symptom, no worse, so far as I have observed, than the opposite and more familiar sign of weakness, too early emission. One of my patients who was making arrangements to get married tried his powers with a strange woman and had this experience of delayed emission, and became much discouraged; but after marriage there was no repetition of this difficulty. Meantime, however, he had taken local and general treatment.

X. *Relaxed Scrotum with or without Varicocele.*—Varicocele is a very common condition, and is not always a sign of serious weakness of the reproductive system as a whole; but as an accompaniment of sexual neurasthenia it is worthy of notice. In its various grades and stages it is found in a very large number of those who have the other symptoms of sexual trouble above described. A larger proportion of such patients have varicocele than of persons who are in perfect health. The direct cause appears to be mainly mechanical inability of the atonic vessels to properly dispose of the blood that flows through them. This condition is satisfactorily relieved by a properly adjusted suspensory bandage ; but this is palliative only, and, as a rule, must be worn all the time, at least during warm weather. We do not usually find varicocele in boys under the age of fourteen; but we do find it appearing as an accompaniment or sequence of the different stages of spermatorrhœa. To one case of pronounced varicocele there are many cases of relaxed scrotum, a sort of wet rag and pendulous condition that is never found in perfect health. Patients complain of this, and say that there is also a sensation of goneness and weakness, of laxity and flabbiness, and even positive pain and neuralgia in those parts. This feeling, which is analogous to the same sensations in the stomach in nervous dyspepsia, disappears as the patient gets stronger. One of the interesting immediate effects of an application of the faradic current to these parts is a contraction of the scrotum, a visible drawing together like a work-bag. This effect may be produced also by faradization of the inner side of the thigh; the so-called cremaster reflex. Excessive sweating of the scrotum, scrotal hyperidrosis, may accompany varicocele. I am not sure whether it does not also exist independently. It is certainly most annoying on the side where the varicocele exists. An unpleasant odor sometimes accompanies this hyperidrosis. This symptom, like the local itching previously spoken of, is made worse temporarily by excess or excitation.

XI. *Transient Attacks of Retention of Urine or of Difficulty of Micturition and Frequent Urination.*—A number of my patients have complained of functional disorders of micturition, which are to be diagnosticated from similar symptoms coming from stricture by their capriciousness and demonstrable dependence on nervous excitation.

Some of these cases have ordinarily no trouble in urinating; but if they chance to be at a public urinal where a line of persons is behind them waiting for their turn, they can do nothing. This is a very good illustration of one of the phases that this function assumes in conditions of debility; it is mainly subjective—mind acting on body—but if the parts were in their full strength the mind would not produce this effect. Their very anxiety to urinate, in order to make way for others, keeps them from doing it.

Analogous illustrations in other functions are very abundant; extreme exertion of the will defeats its object; hysteria and hystero-epilepsy, and especially trance, exemplify this general law in ways innumerable. It is indeed one of the diagnostic features of trance that the subject cannot do the very thing that he particularly wants to do. A sign that a mesmerized subject is under "the influence" and ready for experiment, is the inability to open the eyes when commanded to do so, and when with all his might he tries to do so and fails. There are cases of hysterical paralysis that only recover when we succeed in calling off the patient's attention from the paralyzed limbs to some other portion of the body; the attempt to walk makes it impossible for them to walk.

A patient who has lately consulted me says that he cannot urinate if he is in a room with any one. Sometimes also there is a very frequent desire to urinate; this symptom is both common and annoying, compelling the patient to get up many times during the night.

When the symptoms come from stricture or from contracted meatus or phimosis, they are more likely to be permanent until the source of the irritation is removed.

XII. *Abnormal erectile Excitability.—Diurnal Orgasms.*—One of the most common and interesting of the many phenomena of debility of the reproductive system is exalted reflex excitability. In the reproductive apparatus this is illustrated in a very remarkable way. Erection takes place on the slightest external or subjective irritation; crossing the legs, the friction of the clothing or of manipulation, riding in a carriage or in the cars, the touch of a woman's hand or dress. The subjective or mental reflexes are still more annoying; the sight of a person of the other sex or the thought of them, a mere reference to any subject that suggests sexual matters, no matter how indirectly, may in these cases bring on erection, and even watering of the penis (weeping penis) or flow of the secretions of Cowper's and the prostate glands.

Persons who have this symptom may deceive themselves and suppose that all this excitability means health and vigor, whereas it is but one of the results and signs of debility, oftentimes one of the first that is noticed, inasmuch as it belongs to the earlier and irritable stages of these maladies.

Those who have this symptom are precisely in the condition of those who have certain forms of nervous dyspepsia with abnormal appetite and inability to go long without eating, and a disposition, sometimes beyond control, to eat much more than they can digest or than the system needs.

I have been consulted for a case where there were frequent *orgasms without erection* during the day. Sometimes these orgasms forced the patient to grasp the head of the penis. Under the use of alkalies this symptom disappeared, but in its stead came nocturnal emissions. The patient was married and in middle life.

XIII. *Priapism and spasmodic Contraction.*— Certain cases of *priapism* and *spasmodic contraction* of the penis may perhaps be referred to under this head — the cases of course being excluded that depend on venereal disease, or leucocythemia.*

In some cases many of these local symptoms exist unattended by any very noticeable constitutional symptoms; the patient feels well and vigorous, equal to severe mental and muscular toil, and uncon-

*See Dr. Peabody's article on Priapism, N. Y. Med. Jour., May, 1880.

scious of any loss of power in any part or function save in the reproductive system. The *stronger* the patient is the more likely is the disease to *localize* itself in confining its manifestations to the genital organs, leaving all the rest of the body unaffected.

In the weak and impressible and nervous, on the other hand, even in those who are prominently of the nervous diathesis though not specially nervous, this local weakness and irritability becomes a source of reflex irritation that may affect all or any part of the nervous system, and expressing itself by innumerable phenomena, some of which when taken in connection with each other and in their relation to the local symptoms already spoken of, serve to establish a clear and unmistakable image of the disease to which the term sexual neurasthenia is here applied.

Some of these distant or reflex manifestations are not peculiar to this special form of neurasthenia; they are found in phases of neurasthenia which do not take their root in the reproductive system.

(*To be continued.*)

